# Promoter methylation of *TRIM9* as a marker for detection of circulating tumor DNA in breast cancer patients

**DOI:** 10.1186/s40064-015-1423-7

**Published:** 2015-10-22

**Authors:** Chieko Mishima, Naofumi Kagara, Saki Matsui, Tomonori Tanei, Yasuto Naoi, Masafumi Shimoda, Atsushi Shimomura, Kenzo Shimazu, Seung Jin Kim, Shinzaburo Noguchi

**Affiliations:** Department of Breast and Endocrine Surgery, Osaka University Graduate School of Medicine, 2-2-E10 Yamadaoka, Suita-shi, Osaka 565-0871 Japan

**Keywords:** Breast cancer, *TRIM9*, Methylation, Biomarker

## Abstract

The aim of the present study was to investigate the promoter methylation status of *TRIM9* in breast cancer and to determine the presence of *TRIM9*-methylated circulating tumor DNA (ctDNA) in plasma. Bisulfite sequencing with a next generation sequencer showed *TRIM9* promoter methylation in 92 % (11/12) of breast cancer cell lines (BCCs) and 68 % (13/19) of breast tumor tissues but not in any normal breast tissues (0/19). Methylation ratio of *TRIM9* was significantly lower in basal type (9 %, n = 23) than luminal A (69 %, n = 29, *P* = 0.0003). Quantitative RT-PCR of BCCs disclosed an inverse correlation between *TRIM9* mRNA expression and methylation ratio. *TRIM9* methylated ctDNA in plasma was detected in 18 % (10/56) of metastatic breast cancer patients but not in any of 60 healthy controls. These results indicate that *TRIM9* promoter hypermethylation, which suppresses *TRIM9* mRNA expression, occurs in a significant proportion of breast tumors, and that *TRIM9*-methylated ctDNA thus may serve as a tumor marker for breast cancer.

**Electronic supplementary material:**

The online version of this article (doi:10.1186/s40064-015-1423-7) contains supplementary material, which is available to authorized users.

## Background

The promoter methylation of tumor suppressor genes is one of the most common events in carcinogenesis and has been detected in various malignant diseases including breast cancer. Recent studies have also revealed that tumor-specific gene methylation can be detected in the circulating tumor DNA (ctDNA) of cancer patients and methylated ctDNA is considered to be a promising biomarker. Several genes including *GSTP1 *(*Glutathione S-transferase P1*), *RASSF1A* (*Ras association domain family 1A*), and *RARβ2* (*Retinoic acid receptor β2*) have been identified as methylated genes in breast cancer (Yamamoto et al. 2012; Arai et al. 2006; Miyake et al. 2012) but each of these markers is not always specific to breast cancer and several markers have been used in various combinations. Therefore, the need has arisen for methylation markers which are more specific to breast cancer.

*TRIM9* belongs to the *TRIM* (tripartite motif-containing protein) family which has been identified as an ubiquitin ligase (E3) and plays important roles in various cellular processes (Berti et al. 2002). The *TRIM* family consists of over 70 members, several of which, i.e., *TRIM8, 13, 19, 24, 25, 27, 28, 29, 31, 32, 33, 40* and *69*, are known to be involved in oncogenesis or tumor progression by affecting specific signal pathways such as *RARα* and *p53* (Hatakeyama 2011). Specifically for breast cancers, *TRIM 24, 25* and *27* have been shown to be significant for breast cancer prognosis, such as facilitation of the ubiquitination of estrogen receptors or HER2 gene amplification (Hatakeyama 2011; Tsai et al. 2010; Chambon et al. 2011; Suzuki et al. 2005; Cao et al. 1996). *TRIM9* protein is known as a brain-specific E3 ligase expressed in the human brain neurons and associated with neurological disorders such as Parkinson’s disease, Alzheimer’s disease, epilepsy and stroke (Tanji et al. 2010; Winkle et al. 2014; Shi et al. 2014). However, there have been no reports on possible correlation between *TRIM9* and carcinogenesis.

Using the Illumina Human Methylation 450 database, we found *TRIM9* is specifically methylated in breast cancer tissues. The aim of the present study was therefore first to investigate whether methylation of *TRIM9* promoter is associated with its gene expression in breast cancer cells, and second to clarify the clinicopathological characteristics of *TRIM9* methylated breast tumors. We analyzed the methylation of *TRIM9* promoter by means of next generation sequencing (NGS), which yields a quantitative methylation ratio within a broad CpG area. Lastly, we examined whether *TRIM9* methylated ctDNA can be detected in plasma of breast cancer patients and explored its utility as a novel blood biomarker for breast cancer diagnosis.

## Methods

### Extraction of targeted gene

We used a common methylation database, Illumina Human Methylation 450, provided by the Cancer Genome Atlas (TCGA) Data Portal, National Cancer Institute, Washington, D.C., USA (http://cancergenome.nih.gov/) to find 90 cases which included methylation data of both primary breast carcinoma and normal breast tissue (https://tcga-data.nci.nih.gov/tcga/dataAccessMatrix.htm?mode=ApplyFilter&showMatrix=true&diseaseType=BRCA&tumorNormal=TN&tumorNormal=T&tumorNormal=NT&platformType=2&platformType=42). We downloaded the β-score calculated from about 485,000 CpG sites of 90 paired cancerous and non-cancerous breast tissues, and 547 probes met all of three criteria for inclusion in our study, that is, methylation ratio in cancer tissues >45 %, methylation ratio in normal tissues <5 %, and area under the ROC curve >0.85. Next, we used the *t* test to compare the methylation status of breast cancer and other cancers. Finally, ten probes showing the highest methylation ratio specific to breast cancers qualified as candidates, and among these we decided to target *TRIM9*, since this was the only probe as yet not known to be associated with breast cancers.

### Patients and breast tumor samples

#### Study I

Nineteen pairs of tumor tissues and normal tissues were obtained at surgery between 2001 and 2004 from primary breast cancer patients who had received no preoperative chemotherapy or hormonal therapy. The clinicopathological characteristics of these patients are summarized in Additional file 1: Table S1. Normal tissues were obtained from a quadrant other than the one harboring cancer. Tissue samples were snap frozen in liquid nitrogen and kept at −80 °C until use.

#### Study II

Stage II or III primary breast cancer patients (n = 107), who had been treated with neoadjuvant chemotherapy (NAC) consisting of paclitaxel (80 mg/m^2^) weekly for 12 cycles followed by 5-FU (500 mg/m^2^), epirubicin (75 mg/m^2^) and cyclophosphamide (500 mg/m^2^) every 3 weeks for four cycles at Osaka University Hospital between 2004 and 2009, were retrospectively included in this study. Each patient underwent vacuum assisted biopsy (VAB) of the tumors, and the tumor samples were snap frozen in liquid nitrogen and kept at −80 °C until use. Histological grade, ER, PR, and HER2 status were determined as described in a previous report of ours (Miyake et al. 2012). Ki67 was classified as “high” when ≥20 % of tumor cells were immunohistochemically positive (clone; MIB-1). Pathological complete response (pCR) was defined as no evidence of invasive cancer components in breast irrespective of any axilla lymph nodes metastases. Intrinsic subtypes were determined by means of DNA microarray using the PAM50 method as previously described (Naoi et al. 2011; Parker et al. 2009). The clinicopathological characteristics of these patients are summarized in Table 1. These studies were approved by the Ethical Review Board of Osaka University Hospital and the Research Ethics Committee of Osaka University, and informed consent was obtained from each patient before sampling.

**Table 1:** Comparison of *TRIM9* methylation ratio with various clinicopathological parameters of breast tumors

Characteristics	Total	*TRIM9*	
		Methylation ratio mean ± SE	*P* value*
All cases	107		
Age (years)			
<50	49	11.2 ± 1.54	0.052
≥50	58	7.44 ± 1.10	
Menopausal status			
Pre	51	10.7 ± 1.51	0.117
Post	56	7.73 ± 1.14	
Tumor size			
T1+2	84	8.97 ± 1.04	0.709
T3+4	23	9.83 ± 2.19	
Lymph node metastasis			
Negative	30	10.9 ± 2.27	0.338
Positive	77	8.48 ± 0.96	
Stage			
II	88	9.02 ± 1.02	0.768
III	19	9.75 ± 2.50	
Histological type			
IDC	97	8.66 ± 0.99	0.104
ILC	10	13.9 ± 2.68	
Estrogen receptor			
Negative	42	3.47 ± 0.56	<0.0001
Positive	65	12.8 ± 1.32	
Progesterone receptor			
Negative	65	6.93 ± 1.01	0.005
Positive	42	12.6 ± 1.70	
HER2 receptor			
Negative	76	9.07 ± 1.10	0.893
Positive	31	9.35 ± 1.85	
TNBC			
No	82	11.2 ± 1.13	<0.0001
Yes	25	2.39 ± 0.32	
Subtype (IHC)			
LumA	51	12.3 ± 1.42	<0.0001
LumB	14	14.6 ± 3.40	
HER2	17	5.06 ± 1.21	
TN	25	2.39 ± 0.32	
Subtype (PAM50)			
LumA	29	13.7 ± 1.89	<0.0001
LumB	21	10.1 ± 2.24	
HER2	16	9.90 ± 2.65	
Basal-like	23	3.22 ± 0.84	
Normal-like	18	7.70 ± 2.24	
Histological grade			
1+2	86	9.89 ± 1.09	0.112
3	21	6.12 ± 1.64	
Ki67			
Low (<20 %)	44	10.2 ± 1.31	0.366
High (≥20 %)	62	8.43 ± 1.33	
Unknown	1		
Clinical response			
No CR	70	8.62 ± 1.13	0.434
CR	37	10.2 ± 1.70	
Histological response			
Grade 1, 2a	58	11.4 ± 1.37	0.006
Grade 2b, 3	49	6.45 ± 1.17	
Pathological response			
No pCR	74	11.0 ± 1.20	0.001
Pcr	33	5.02 ± 1.15	
Recurrence			
No	90	9.88 ± 1.08	0.007
Yes	17	5.30 ± 1.12	

* t test

### DNA extraction and sodium bisulfite treatment

Total DNA from cell lines was isolated using TRIzol^®^ reagent (Invitrogen, Carlsbad, CA, USA) and total DNA from the breast tissues was extracted using the DNeasy^®^ Blood and Tissue Kit (QIAGEN, Valencia, CA, USA). 1 μg of genomic DNA was then subjected to sodium bisulfite treatment with the EpiTect^®^ Bisulfite Kit (QIAGEN), and the QIAamp^®^ Circulating Nucleic Acid Kit (QIAGEN) was used to extract plasma DNA from a 2 ml plasma sample, which was then subjected to sodium bisulfite treatment as previously described (Fujita et al. 2012).

### Quantitative *TRIM9* promoter methylation analysis using NGS

**Fig. 1:**
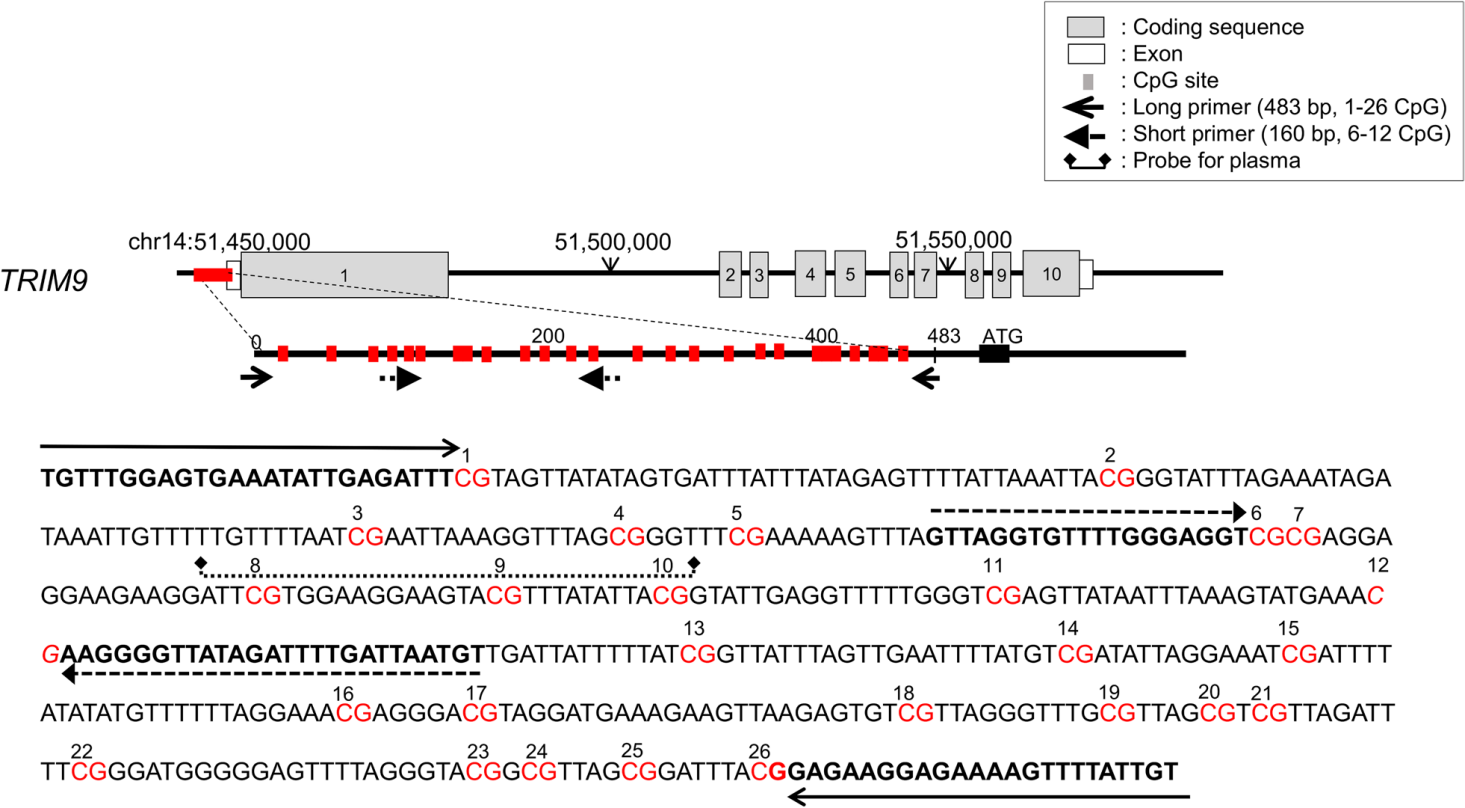
Primer designs for DNA methylation analysis of *TRIM9* using NGS and for detection of *TRIM9* methylated ctDNA using real-time PCR. Long primer sets were designed for DNA methylation analysis of *TRIM9* by means of NGS (*solid arrow*). *TRIM9* methylated ctDNA in plasma was detected by means of real-time PCR using the short primers (*dashed arrow*) and probes (*dotted line*)

The NGS methylation assay was performed with the GS Junior system (Roche Diagnostics, Basel, Switzerland) according to the manufacturer’s instructions, and data was analyzed with GS Amplicon Variant Analyzer (AVA) software (version 2.7; Roche Diagnostics). The methylation index (MI) was calculated by dividing the number of cytosines by that of the total reads at each CpG site. NGS primers used for *TRIM9* methylation of frozen tissues or cell lines were designed as follows: forward 5′-TGTTTGGAGTGAAATATTGAGATTT-3′, reverse 5′-ACAATAAAACTTTTCTCCTTCTCC-3′ (long primer; Fig. 1). The average methylation ratio of 12 of the 26 CpG sites (6th–17th CpG), which showed the most significant difference between cancer and normal tissues, was used for methylation analysis. NGS primers used for DNA from formalin-fixed paraffin embedded (FFPE) specimens were designed as follows: forward 5′-AGTTTAGTTAGGTGTTTTGGGAGGT-3′, reverse 5′-ACATTAATCAAAATCTATAACCCCTTC-3′ (short primer; Fig. 1). The NGS short primer included 7 CpG sites, corresponding to 6th–12th CpG.

### In situ hybridization (ISH) for *TRIM9* mRNA and immunohistochemical staining (IHC) for *TRIM9* protein

The QuantiGene^®^ViewRNA ISH Tissue Assay kit (Affymetrix, Santa Clara, CA, USA) was used according to the manufacturer’s protocol. FFPE Sections (4 μm) of tumor tissues were incubated at 98 °C with a pretreatment solution for 20 min, followed by protease digestion for 10 min. The *TRIM9*-specific View RNA™ Probe set (Affymetrix) was hybridized for 2 h. A *TRIM9* specific probe set was then designed to hybridize the common sequence of *TRIM9_v1* and *TRIM9_v2* (1319 bp). ISH images were obtained under fluorescent microscopy (BZ9000; Keyence, Osaka, Japan). Signal intensity was semi-quantitatively determined based on the number of cytoplasmic fluorescent dots in five non-overlapping fields at high-power magnification (×400).

Formalin-fixed paraffin Sections (3 μm) of the tumor tissues were obtained for immunohistochemical staining with rabbit anti-*TRIM9* polyclonal antibody (ProteinTech Group, Inc., Chicago, IL, USA) at a dilution of 1:400 according to a previously described method for ER, PR and Ki-67, with a slight modification in that antigen retrieval was accomplished by incubating at 98 °C in citrate buffer (pH 9.0) for 40 min (Shimomura et al. 2009; Tanei et al. 2009). Immunohistochemical staining for *TRIM9* was classified as 3+ (strongly positive), 2+ (intermediately positive), 1+ (weakly positive) or 0 (negative). The sections were counterstained with hematoxylin.

### Isolation of breast tumor cells by magnetic-activated cell sorting (MACS)

Breast tumor cells were separated from the FFPE tumor tissues with the magnetic-activated cell sorting (MACS) method using the EasySep Human EpCAM Positive Selection Cocktail, the EasySep Human MUC1 Positive Selection Cocktail and EasySep Magnetic Particles (Stem Cell Technologies, Vancouver, BC, Canada) as previously described (Otani et al. 2014). Total DNA was extracted from these isolated tumor cells using the QIAamp^®^ DNA FFPE Tissue Kit (QIAGEN).

### Demethylation study of cell lines using 5-aza-2′-deoxycytidine

Twelve breast cancer cell lines (BCCs) and one normal breast cell line were cultured under the conditions shown in Additional file 2: Table S2. For demethylation studies, the cultured cells were treated with 10 μmol/L 5-aza-2′-deoxycytidine (5-aza; Sigma-Aldrich, St Louis, MO, USA) or with dimethylsulfoxide (DMSO) as control for 72 h, with the medium changed every 24 h.

### RNA extraction and real-time qRT-PCR

Total RNA was isolated from cell lines using TRIzol^®^ reagent (Invitrogen), and 1 μg of total RNA was reverse-transcribed for single strand cDNA, using random primers and the ReverTra Ace^®^ qPCR RT kit (Toyobo, Osaka, Japan). Reverse-transcription reaction was performed first at 65 °C for 5 min and then at 37 °C for 15 min and at 98 °C for 5 min. Quantitative mRNA expression was measured using the Light Cycler 480 Real-time PCR System (Roche Applied Science, Mannheim, Germany) at 95 °C (10 min), followed by 50 cycles at 95 °C (15 s) and at 60 °C (60 s), and 1 cycle at 50 °C (10 s). *TRIM9* and *glyceraldehyde 3*-*phosphate dehydrogenase* (*GAPDH*) TaqMan^®^ Gene Expression Assays (assay identification numbers: Hs00364838_m1 and Hs02758991_g1. Applied Biosystems, Foster City, CA, USA) were used for the real time qPCR assay. The expression of *TRIM9* was normalized to that of *GAPDH*, and each assay was performed in duplicate. For the 5-aza treated BCCs, each treated cell line was normalized to the value of its control, which was set at 1.

### Measurement of *TRIM9* methylated ctDNA in plasma

Two ml of plasma samples was obtained from healthy controls (n = 60) and from metastatic breast cancer (MBC) patients (n = 56), 41 cases of recurrent and 15 of primary advanced breast cancer, before they had been treated at Osaka Police Hospital or Osaka University Hospital between 2012 and 2014. *TRIM9* methylated ctDNA in plasma was measured by using quantitative methylation-specific PCR (MSP) with the *TRIM9* short primer (Fig. 1). The double-dye probe, including *TRIM9* 8th–10th CpG sites (5′-TCGTGGAAGGAAGTACGTTTATATTAC-3′; Fig. 1) for detection of *TRIM9* methylated ctDNA in plasma, is shown in Fig. 1. 9 µl aliquot each of the bisulfite DNA, eluted for a total PCR reaction volume of 20 µl, was placed in 96-well plates for the *TRIM9* PCR reactions. *TRIM9* methylated ctDNA in plasma was classified as positive when quantification cycles were less than 50 cycles for *TRIM9*.

### Statistical analysis

The JMP statistical software package (version 11.2.1; SAS Institute, Cary, NC, USA) was used for statistical analyses. Association between the various parameters and *TRIM9* methylation ratio was evaluated using the *t* test for two groups or the Kruskal–Wallis test for more than two groups. The paired *t* test was used for comparison of frozen cancer and normal tissue MI in matched-pair samples. The Tukey test was used for comparison of the *TRIM9* methylation ratio for each subtype. The univariate and multivariate analysis of various parameters for the association with pCR were performed with the logistic regression model. All statistical analyses were two-sided and *P* values <0.05 were considered to be statistically significant.

## Results

### Promoter methylation of *TRIM9* and its impact on gene expression in BCCs

**Fig. 2:**
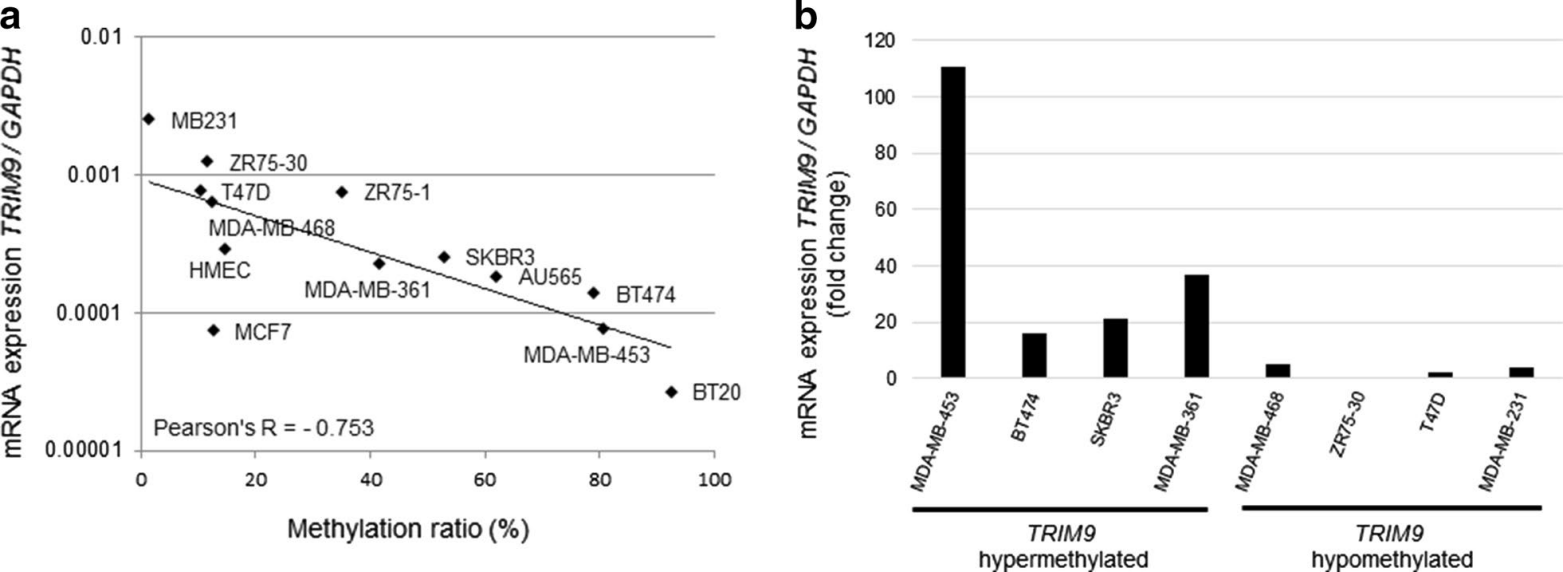
*TRIM9* methylation status and mRNA expression of 13 breast cancer cell lines. **a** Correlation between *TRIM9* methylation index and mRNA expression. **b** Fold changes in *TRIM9* mRNA expression of 8 cell lines after 5-aza treatment

To study the methylation status of *TRIM9*, we performed an NGS methylation assay of the *TRIM9* promoter in 12 BCCs and a normal breast cell line (HMEC). The methylation ratio of the *TRIM9* gene promoter varied greatly from 10.3 to 92.6 % in 11 of the BCCs and was relatively hypomethylated in the HMEC cells (Fig. 2a; Additional file 3: Table S3).

We next used *TRIM9*-specific primers and probes to investigate the *TRIM9* mRNA expression by qRT-PCR. An inverse correlation between the *TRIM9* mRNA expression and methylation ratio was clearly observed (Pearson’s correlation coefficient: −0.753) (Fig. 2a). We then treated eight of these cell lines with a demethylating reagent (10 μM 5-aza) and compared the mRNA expression of the treated and untreated cells. 5-aza treatment induced a 16- to 110-fold up-regulation of mRNA expression in all four hypermethylated BCCs (MDA-MB-453, BT474, SKBR3 and MDA-MB-361), while no up-regulation was detected in any of the four hypomethylated BCCs (MDA-MB-468, ZR75-30, T47D and MDA-MB-231), demonstrating that the *TRIM9* gene was re-expressed by the demethylation of its promoter region (Fig. 2b).

### Methylation and expression of *TRIM9* in human breast cancer tissues

**Fig. 3:**
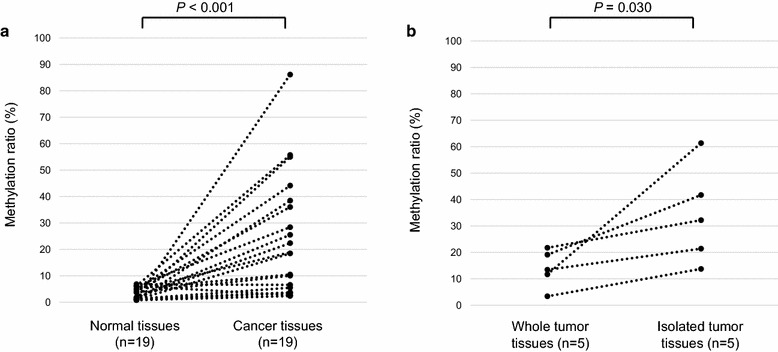
Methylation status of *TRIM9* in breast cancer and normal breast tissues. **a** Comparison of *TRIM9* methylation index for 19 paired normal breast and cancer tissues. **b** Comparison of *TRIM9* methylation index for whole breast cancer tissues and tumor cells isolated with the MACS method

To study the methylation status of *TRIM9* in human breast cancer and normal breast tissues, we performed an NGS methylation assay using the 19 paired tumor and normal tissues (study I). The methylation ratio was significantly higher for the tumor tissues than the normal tissues (median values, 19 and 1.8 %, respectively, *P* = 0.00067; Fig. 3a). The ratio of *TRIM9* hypermethylated tumors (methylation ratio ≥8.2 %) was 68 %.

For a more accurate assessment of the cancer cell-specific methylation status, we isolated the tumor cells from the FFPE tumor tissues with the MACS method, and the isolated tumor cells were then subjected to an NGS methylation assay. Five tumor tissues with a low methylation ratio (<25 %) were further analyzed since it was thought the low methylation ratio of some of them was due to contamination by the normal stromal and inflammatory cells. Methylation ratios increased in the tumor cells isolated from whole tumor tissues (Fig. 3b).

### Relationship between *TRIM9* methylation and clinicopathological characteristics

**Fig. 4:**
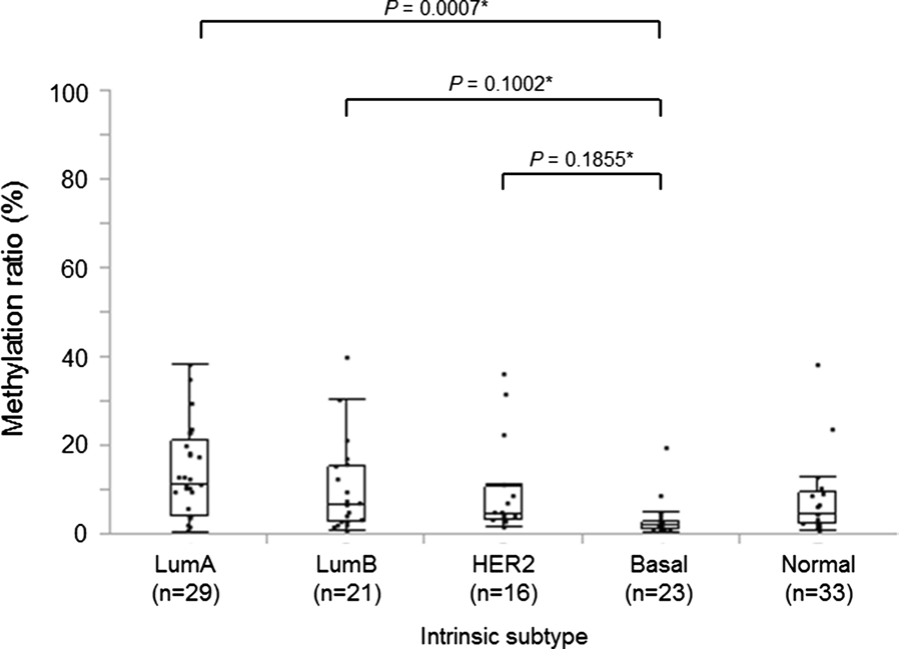
Methylation status of *TRIM9* in 107 breast cancer tissues. Breast tumors were classified into five intrinsic subtypes (luminal A, luminal B, HER2, basal-like, normal breast-like) by PAM50 for comparison of their methylation index of *TRIM9*. *Tukey’s test

An NGS methylation assay of *TRIM9* was performed using the biopsy specimens obtained before NAC (study II) to examine the relationship between *TRIM9* methylation and the various clinicopathological parameters including response to NAC (Table 1). *TRIM9* hypermethylation (methylation ratio ≥8.2 %) was observed in 40 % (43/107) of the specimens. *TRIM9* hypermethylation was significantly associated with ER positivity, PR positivity, low histological grade and no pCR (Table 1). Furthermore, the methylation ratio was significantly lower for basal type (9 %) than for luminal A type (*P* = 0.0007; Fig. 4).

**Fig. 5:**
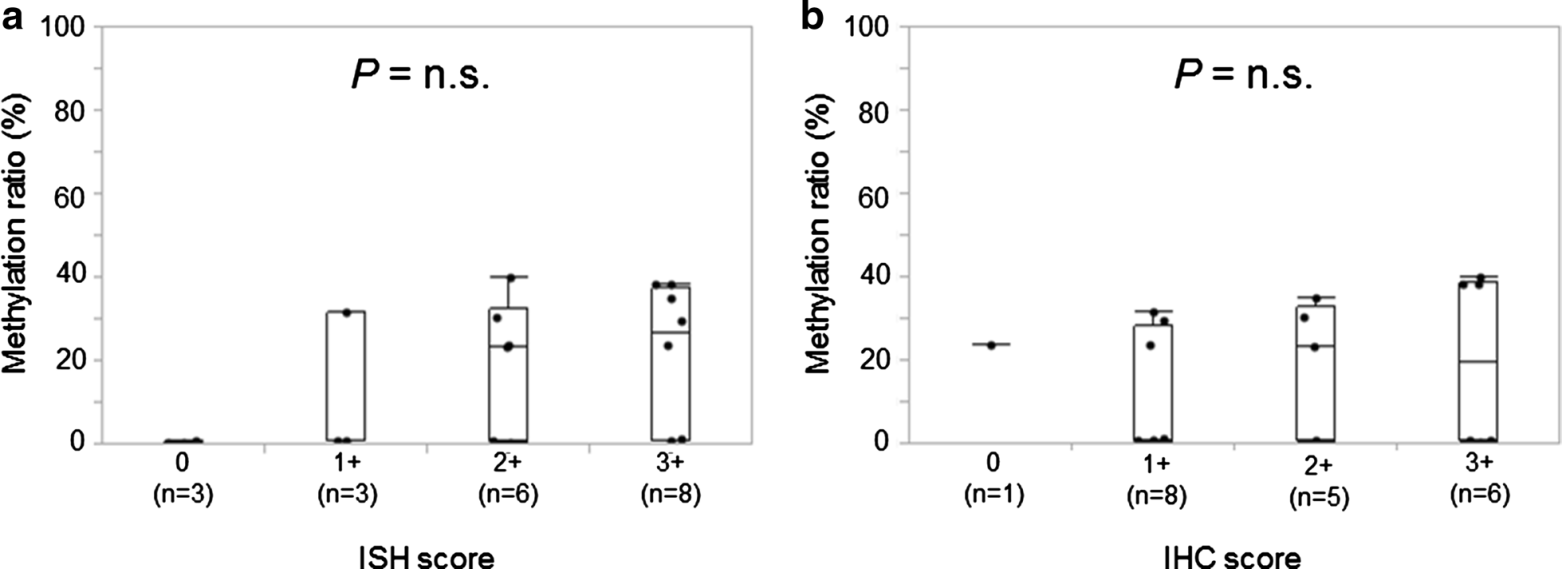
Association between *TRIM9* mRNA expressions obtained with ISH and IHC analysis and methylation ratios in breast cancer tissues. *TRIM9* hypermethylated or hypomethylated breast cancer tissues were subjected to ISH (a) and IHC (b) for *TRIM9* mRNA. Each immunoreactivity was one of four scores (0, 1+, 2+ and 3+). ISH, in situ hybridization; IHC, immunohistochemistry

Next, to determine whether methylation is related to gene expression, we subjected the *TRIM9* hypermethylated (n = 10) and hypomethylated tumors (n = 10) to ISH and IHC and found that neither ISH signals nor IHC scores in tumor cells were significantly associated with methylation ratios (Fig. 5).

### Relationship between *TRIM9* methylation and response to NAC

The clinicopathological parameters were assessed by means of univariate analysis for their association with pCR (Table 2). Age, Ki67, ER, PR, HER2, and *TRIM9* methylation were found to be significantly associated with pCR. The multivariate analysis showed that only ER, but not *TRIM9* methylation, was a significant and independent predictor for pCR.

**Table 2:** Univariate and multivariate analysis of clinicopathological factors for pCR

Characteristics	Univariate analysis			Multivariate analysis		
	Odds ratio	95 % CI	*P* value	Odds ratio	95 % CI	*P* value
Age (≥50 vs <50)	3.13	1.29–7.66	0.0091	2.24	0.80-6.56	0.1236
T stage (T1+2 vs T3+4)	1.80	0.61–5.35	0.2743			
Lymph node status (positive vs negative)	1.68	0.64–4.41	0.2858			
ER (negative vs positive)	10.5	4.00–27.4	<0.0001	5.14	1.23–27.2	0.0240
PgR (negative vs positive)	5.60	1.95–16.1	0.0004	0.98	0.17–4.86	0.9823
HER2 (positive vs negative)	2.47	1.03–5.94	0.0440	1.98	0.69–5.76	0.2002
*TRIM9* methylation (<8.2 vs ≥8.2 %)	10.6	2.96–37.6	<0.0001	3.96	0.97–20.1	0.0545

*CI* confidence interval

### Detection of *TRIM9*-methylated ctDNA in MBC patients

*TRIM9*-methylated ctDNA in plasma of 56 MBC patients and 60 healthy controls was assayed by using MSP. An amplification curve of the eight standards was obtained by diluting the methylated human control DNA (diluted to 10, 3, 1, 0.3, 0.1, 0.03, 0.01 and 0 ng/ml plasma). The limit of detection for methylated *TRIM9* DNA was 0.1 ng/ml in plasma. *TRIM9* methylated ctDNA was detected in 18 % (10/56) of MBC patients but not in any of the healthy controls. Primary breast tumor tissues for determination of *TRIM9* methylation status were available for 27 of the 56 cancer patients, and *TRIM9* methylated ctDNA was detected in 44 % (4/9) of the MBC patients with *TRIM9* hypermethylated tumors but in only 6 % (1/18) of the MBC patients with *TRIM9* hypomethylated tumors (Table 3).

**Table 3:** Sensitivity for detection of methylated *TRIM9* in plasma of Stage IV and metastatic breast cancer patients

	Total	Methylated *TRIM9* in plasma			
		Positive		Negative	
		No.	(%)	No.	(%)
Healthy control	60	0	(0)	60	(100)
MBC patients (total)	56	10	(18)	46	(82)
MBC patients with *TRIM9* hypermethylated tumors	9	4	(44)	5	(56)
MBC patients with *TRIM9* hypomethylated tumors	18	1	(6)	17	(94)

*MBC* metastatic breast cancer

## Discussion

For this study, we selected the *TRIM9* gene as a breast cancer specific methylation marker by referring to the methylation array database and observed *TRIM9* hypermethylation in 92 % (11/12) of the BCCs and 68 % (13/19) of breast tumor tissues but not in any of the normal breast epithelial cell line (HMEC) cells or normal breast tissues. Several methylation markers for breast cancers have been investigated, such as *GSTP1*, *RASSF1A* and *RARβ2*, and hypermethylation of these genes has been reported as, respectively, 17–48 %, 43–90 % and 26–78 % in breast cancer tissues and as 2–3 %, 3–8 % and 0 % in normal breast tissues (Yamamoto et al. 2012; Jung et al. 2013; Hagrass et al. 2014; Pirouzpanah et al. 2015), indicating the equally high sensitivity and specificity of *TRIM9* as a methylation marker for breast cancer. Although methylation of these other genes has reportedly been detected in other types of cancers than breast cancer (Zhang et al. 2015; Li et al. 2015a, b; Grote et al. 2005), *TRIM9* is methylated specifically in breast cancer according to the Illumina Human Methylation 450 database (http://cancergenome.nih.gov/), implying that *TRIM9* may function as a methylation marker that is specific to breast cancer. The fact that the methylation ratio was lower in tumor tissues than BCCs seems to be explained by the contamination of tumor tissues by the normal stromal and inflammatory cells, since the tumor cells isolated by MACS showed an evidently higher methylation ratio than the tumor tissues from which they derived. These results indicate that breast tumor cells, but not normal breast epithelia, actually harbor *TRIM9* methylation.

We found that *TRIM9* mRNA expression correlated inversely with *TRIM9* methylation ratio in BCCs, and that treatment of *TRIM9* hypermethylated BCCs with a demethylating reagent resulted in the reactivation of *TRIM9* mRNA expression. Although these findings suggest that *TRIM9* expression is epigenetically regulated by promoter methylation in BCCs, we could not confirm the occurrence of such an epigenetic regulation in breast tumor tissues. No reports have been published so far on possible associations between promoter methylation and gene expression in the *TRIM* family, including *TRIM9*. *TRIM9* is known to be up-regulated by interferons, suggesting that another mechanism than promoter methylation may be more important in the regulation of gene expression in breast cancer tissues (Carthagena et al. 2009) although promoter methylation seems to play a significant role in vitro as we have shown in the present study.

The *TRIM9* methylation ratio was significantly lower in basal type tumor than in the other intrinsic subtypes, which is consistent with the report that basal type tumors are more globally hypomethylated than the other subtypes (Cancer Genome Atlas Network 2012). Although *TRIM9* hypermethylation was found to be significantly associated with no pCR, this does not necessarily mean that *TRIM9* hypermethylation plays a significant role in resistance to chemotherapy. Multivariate analysis failed to demonstrate any statistical significance for *TRIM9* hypermethylation as an independent predictor for no pCR. It is thus speculated that *TRIM9* hypermethylation may be indirectly associated with no pCR via its strong association with ER, which is a well-established predictor for no pCR (Carey et al. 2007; Rouzier et al. 2005; Ignatiadis and Sotiriou 2013). Putting these considerations together suggests that *TRIM9* is unlikely to play a significant role in chemotherapy resistance or is at least, not a clinically useful predictor for no pCR.

Our study detected *TRIM9* methylated ctDNA in only 18 % (10/56) of MBC patients. However, this sensitivity was as high as 44 % (4/9) when only the MBC patients with *TRIM9* methylated tumors were taken into consideration, but it was only 5.6 % (1/18) for those without *TRIM9* methylated tumors. Previous studies have reported that aberrant promoter methylation in serum DNA of MBC patients was 18–25 % for *GSTP1* (Yamamoto et al. 2012; Müller et al. 2003; Sharma et al. 2010), 33–39 % for *RASSF1A* (Yamamoto et al. 2012; Müller et al. 2003; Kim et al. 2010) and 20–87 % for *RARβ2* (Yamamoto et al. 2012; Sharma et al. 2010; Kim et al. 2010). Although methylated *TRIM9* in blood was less sensitive than the existing methylation markers, the specificity was 100 % which was superior to that of any other genes (2–10 % for *GSTP1*, 0–10 % for *RASSF1A* and 5–6 % for *RARβ2*) (Yamamoto et al. 2012; Müller et al. 2003; Kim et al. 2010). *TRIM9*-methylated ctDNA may thus be a potential tumor marker and might work better in combination with other blood biomarkers for breast cancers to compensate for its lower sensitivity. However, the number of patients in our study was limited and further prospective studies are needed to verify our findings.

## Conclusions

We found that *TRIM9* promoter hypermethylation occurred in 68 % of breast tumors but not in normal breast tissues. Methylated *TRIM9* was detected in the plasma from 44 % of metastatic breast cancer patients with *TRIM9* methylated tumors. Although the regulatory mechanism of *TRIM9* gene expression and its biological functions remain unclear, our preliminary results suggest that methylated *TRIM9* may serve as a novel blood biomarker specific to breast cancer patients.

## Additional files

10.1186/s40064-015-1423-7 Clinicopathological characteristics of breast tumors used for comparison of *TRIM9* methylation index for paired tumor and normal breast tissues.
10.1186/s40064-015-1423-7 Breast cell lines used in this study.
10.1186/s40064-015-1423-7 *TRIM9* methylation index and mRNA expression in breast cancer cell lines.

## Abbreviations

ER: Estrogen receptor
PR: Progesterone receptor
HER2: Human epidermal growth factor receptor 2
CEA: Carcinoembryonic antigen
CA15-3: Carbohydrate antigen15-3
