# Supplementary material for: Promoter methylation of TRIM9 as a marker for detection of circulating tumor DNA in breast cancer patients
Source: Springerplus. 2015 Oct 22;4:635. doi: 10.1186/s40064-015-1423-7 (PMC4627990; doi:10.1186/s40064-015-1423-7)
Supplement: Supplementary file 1 — 10.1186/s40064-015-1423-7 Clinicopathological characteristics of breast tumors used for comparison of TRIM9 methylation index for paired tumor and normal breast tissues. [file 40064_2015_1423_MOESM1_ESM.doc]

Table S1. Clinicopathological characteristics of breast tumors used for comparison of *TRIM9* methylation index for paired tumor and normal breast tissues

| Characteristics |  | No. of patients | % |
| --- | --- | --- | --- |
| All cases |  | 19 |  |
| Age (years) | < 50 | 9 | 52.6 |
|  | ≧ 50 | 10 | 47.4 |
| Menopausal status | Pre | 10 | 52.6 |
|  | Post | 9 | 47.4 |
| Tumor size | ≦ 2cm | 5 | 26.3 |
|  | > 2cm | 14 | 73.7 |
| Lymph node metastasis | Negative | 13 | 68.4 |
|  | Positive | 6 | 31.6 |
| Histological type | IDC | 18 | 94.7 |
|  | Special type | 1 | 5.26 |
| Histological grade | 1+2 | 13 | 68.4 |
|  | 3 | 6 | 31.6 |
| ER | Negative | 7 | 36.8 |
|  | Positive | 12 | 63.2 |
| PR | Negative | 10 | 52.6 |
|  | Positive | 9 | 47.4 |
| HER2 | Negative | 12 | 63.2 |
|  | Positive | 7 | 36.8 |
| Ki67 | Low | 5 | 26.3 |
|  | High | 1 | 5.26 |
|  | Unknown | 13 | 68.4 |
| Histological type | IDC | 18 | 94.7 |
|  | Special type | 1 | 5.26 |
| Subtype (IHC) | Luminal A^a^ | 9 | 47.4 |
|  | Luminal B^b^ | 3 | 15.8 |
|  | HER2^c^ | 4 | 21.1 |
|  | Triple negative^d^ | 3 | 15.8 |

IDC, invasive ductal carcinoma; IHC, immunohistochemistry; ^a^, ER and/or PR positive, HER2 negative; ^b^, ER and/or PR positive, HER2 positive; ^c^, ER and PR negative, HER2 positive; ^d^, ER ,PR and HER2 negative
