# Supplementary material for: Promoter methylation of TRIM9 as a marker for detection of circulating tumor DNA in breast cancer patients
Source: Springerplus. 2015 Oct 22;4:635. doi: 10.1186/s40064-015-1423-7 (PMC4627990; doi:10.1186/s40064-015-1423-7)
Supplement: Supplementary file 2 — 10.1186/s40064-015-1423-7 Breast cell lines used in this study. [file 40064_2015_1423_MOESM2_ESM.doc]

Table S2. Breast cell lines used in this study

| Cell line | ER | PR | HER2 | Source | Culture medium | Culture condition |
| --- | --- | --- | --- | --- | --- | --- |
| MCF7 | + | + | - | Pleural effusion | DMEM  10%FBS, antibiotics | 37°C, 5%CO2 |
| ZA75-1 | + | + | - | Ascites fluid | RPMI1640  10%FBS, antibiotics | 37°C, 5%CO2 |
| T47D | + | + | - | Pleural effusion | RPMI1640,  10%FBS, antibiotics | 37°C, 5%CO2 |
| ZR75-30 | + | + | + | Ascites fluid | RPMI1640  10%FBS, antibiotics | 37°C, 5%CO2 |
| MDA-MB-361 | + | + | + | Primary breast | DMEM/F12  10%FBS, antibiotics | 37°C, 5%CO2 |
| BT474 | + | + | + | Primary breast | DMEM  10%FBS, antibiotics | 37°C, 5%CO2 |
| SKBR3 | - | - | + | Pleural effusion | McCoy’s 5a  10%FBS, antibiotics | 37°C, 5%CO2 |
| AU565 | - | - | + | Pleural effusion | RPMI1640  10%FBS, antibiotics | 37°C, 5%CO2 |
| MDA-MB-453 | - | - | + | Pleural effusion | DMEM/F12  10%FBS, antibiotics | 37°C, 5%CO2 |
| MDA-MB-231 | - | - | - | Pleural effusion | DMEM/F12  10%FBS, antibiotics | 37°C, 5%CO2 |
| MDA-MB-468 | - | - | - | Pleural effusion | DMEM/F12  10%FBS, antibiotics | 37°C, 5%CO2 |
| BT20 | - | - | - | Primary breast | DMEM  10%FBS, antibiotics | 37°C, 5%CO2 |
| HMEC |  |  |  | Normal breast | MEGM BulletKit® | 37°C, 5%CO2 |
