# Supplementary material for: Promoter methylation of TRIM9 as a marker for detection of circulating tumor DNA in breast cancer patients
Source: Springerplus. 2015 Oct 22;4:635. doi: 10.1186/s40064-015-1423-7 (PMC4627990; doi:10.1186/s40064-015-1423-7)
Supplement: Supplementary file 3 — 10.1186/s40064-015-1423-7 TRIM9 methylation index and mRNA expression in breast cancer cell lines. [file 40064_2015_1423_MOESM3_ESM.doc]

Table S3. *TRIM9* methylation index and mRNA expression in breast cancer cell lines

|  | *TRIM9* methylation ratio (%) | Relative mRNA expression |
| --- | --- | --- |
|  |  | (*TRIM9 / GAPDH*) |
| MCF7 | 12.54833 | 7.44E-05 |
| ZR75-1 | 34.97917 | 0.000764 |
| T47D | 10.31083 | 0.000767 |
| ZR75-30 | 11.535 | 0.001251 |
| MDA-MB-361 | 41.6975 | 0.000232 |
| BT474 | 79.0975 | 0.000139 |
| SKBR3 | 52.795 | 0.000254 |
| AU565 | 61.98417 | 0.000184 |
| MDA-MB-453 | 80.65917 | 7.71E-05 |
| MDA-MB-231 | 1.409167 | 0.00255 |
| MDA-MB-468 | 12.28583 | 0.000633 |
| BT20 | 92.55083 | 2.7E-05 |
| HMEC | 14.55583 | 0.000293 |
